# Transanal endoscopic microsurgery was associated with higher recurrence rate in both low- and high-risk T1 rectal cancer compared to surgical resection

**DOI:** 10.1007/s00464-026-12926-y

**Published:** 2026-06-05

**Authors:** Emelie Nilsson, Lisa Arvidsson, Carl-Fredrik Rönnow, Henrik Thorlacius

**Affiliations:** Department of Clinical Sciences, Lund University. Division of Surgery, Skåne University Hospital, 20502 Malmö, Sweden

**Keywords:** Rectal cancer, Transanal endoscopic microsurgery, Surgical resection, Recurrence

## Abstract

**Background:**

Optimal treatment of T1 rectal cancer (RC) remains to be determined. The aim of this study was to compare recurrence rates after transanal endoscopic microsurgery (TEM) and surgical resection according to risk groups.

**Material and methods:**

This registry-based nationwide cohort study collected data on patients diagnosed with pT1 RC undergoing TEM or surgical resection (January 2009–December 2022) from the Swedish Colorectal Cancer Registry. Differences in recurrence rates were compared using Chi-square and log-rank tests, and were further analyzed using Cox- and logistic regression models.

**Results:**

A total of 228 and 631 patients undergoing TEM and surgical resection, respectively, with a median follow-up time of 59.7 months were identified. Overall recurrence rates were 11% in patients undergoing TEM and 5% following surgical resection, which was reflected in a lower five-year disease-free interval after TEM compared to surgery (88 vs. 95%, *p* = 0.002), with a HR of 2.83 (95% CI 1.56–5.13) after adjusting for sex, age at diagnosis, ASA class, LVI, PNI, mucinous subtype, submucosal invasion, and resection margin. The overall recurrence was higher following TEM compared to surgical resection in both low-risk (OR 5.31, 95% CI 1.10–25.56) and high-risk groups (OR 2.39, 95% CI 1.22–4.66). This corresponded to higher local recurrence rates after TEM compared to surgical resection in patients with high-risk tumors (8 vs. 1%, OR 6.96, 95% CI 2.40–20.17) and low-risk tumors (8 vs. 0%, *p* = 0.016, logistic regression not applicable), whereas no difference in distant recurrence rates was observed between the groups.

**Conclusion:**

This nationwide registry-based cohort study showed that the local recurrence rate after TEM was significantly higher than after surgical resection, even in low-risk pT1 RC. These findings highlight the need for increased awareness of the risk of recurrence after TEM in early RC.

**Graphical abstract:**

**Figure:**
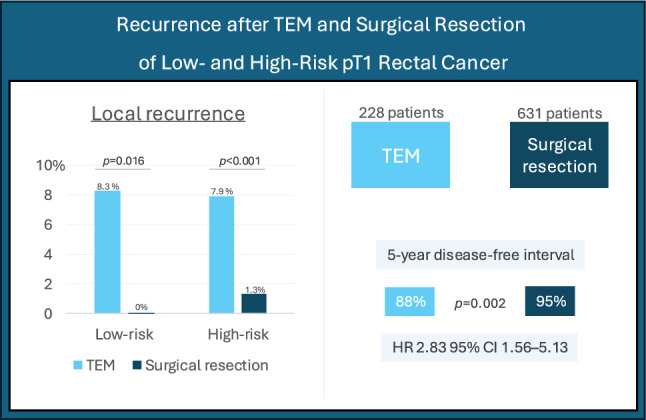

**Supplementary Information:**

The online version contains supplementary material available at 10.1007/s00464-026-12926-y.

Surgical resection has long been considered the gold standard treatment for patients with pT1 rectal cancer (RC) offering excellent oncological results [1]. Conversely, this extensive procedure is associated with high risk of significant complications, such as mortality, anastomotic leakage, permanent stoma, and sexual and urinary dysfunction, with severe impact on long-term quality of life [2–7].

The interest in treating patients with T1 RC using local resection has increased due to technical advances and a higher detection rate of early RC following the implementation of screening programs [8]. With an increasing number of patients diagnosed at an earlier stage, the urgency to define optimal treatment for non-metastasized T1 RC is growing. Transanal endoscopic microsurgery (TEM) is one of several local resection techniques [8–10] and is associated with fewer complications, shorter surgical time, and shorter hospital stay compared to surgical resection [7, 11, 12]. Earlier studies have nonetheless indicated higher local recurrence rates compared to surgery [11–14].

The European Society of Gastrointestinal Endoscopy (ESGE) categorize locally resected tumors into high or low risk, based on histopathological features associated with the risk of lymph node metastases (LNM), including incomplete resection (R1/Rx resection), deep submucosal invasion (> Sm1), lymphovascular invasion (LVI), tumor budding (Bd2–3), and high histologic grade [9]. When adhering to current guidelines, patients with high-risk tumors are generally recommended completion surgical resection, while TEM may be considered definitive treatment for patients with low-risk tumors [9, 15]. However, studies comparing recurrence rates after TEM and surgical resection in T1 RC have rarely incorporate current high- and low-risk criteria [16].

In addition, it is important to recognize that current guidelines are based on the risk of concomitant LNM rather than recurrence, although recurrence is arguably the more clinically relevant outcome for RC patients following treatment.

Based on these considerations, this study aimed to determine whether recurrence rates in T1 RC patients differed between TEM and surgical resection across risk groups.

## Material and methods

### Study design

This is a registry-based retrospective multicenter nationwide cohort study on prospectively collected data. The study is completed following STROBE guidelines. The STROBE checklist is available as Supplementary Table 1.

### Swedish colorectal cancer registry

This study used information from the Swedish Colorectal Cancer Registry (SCRCR), a nationwide quality registry established in 1995. The registry prospectively collects clinical data from the time of cancer diagnosis, including preoperative details such as neoadjuvant treatment, tumor characteristics and follow-up. For surgically resected patients follow-up is routinely performed at 30 days, and at 1, 3, and 5 years postoperatively. Computed tomography (CT) of the abdomen and thorax and CEA is performed on all follow-up occasions, while colonoscopy is performed after three years [17]. Patients treated with local resection undergo standard follow-up, with the addition of repeated endoscopic surveillance [17]. The SCRCR has been shown to achieve an average completeness of 98.8% [18].

### Study population

**Fig. 1:**
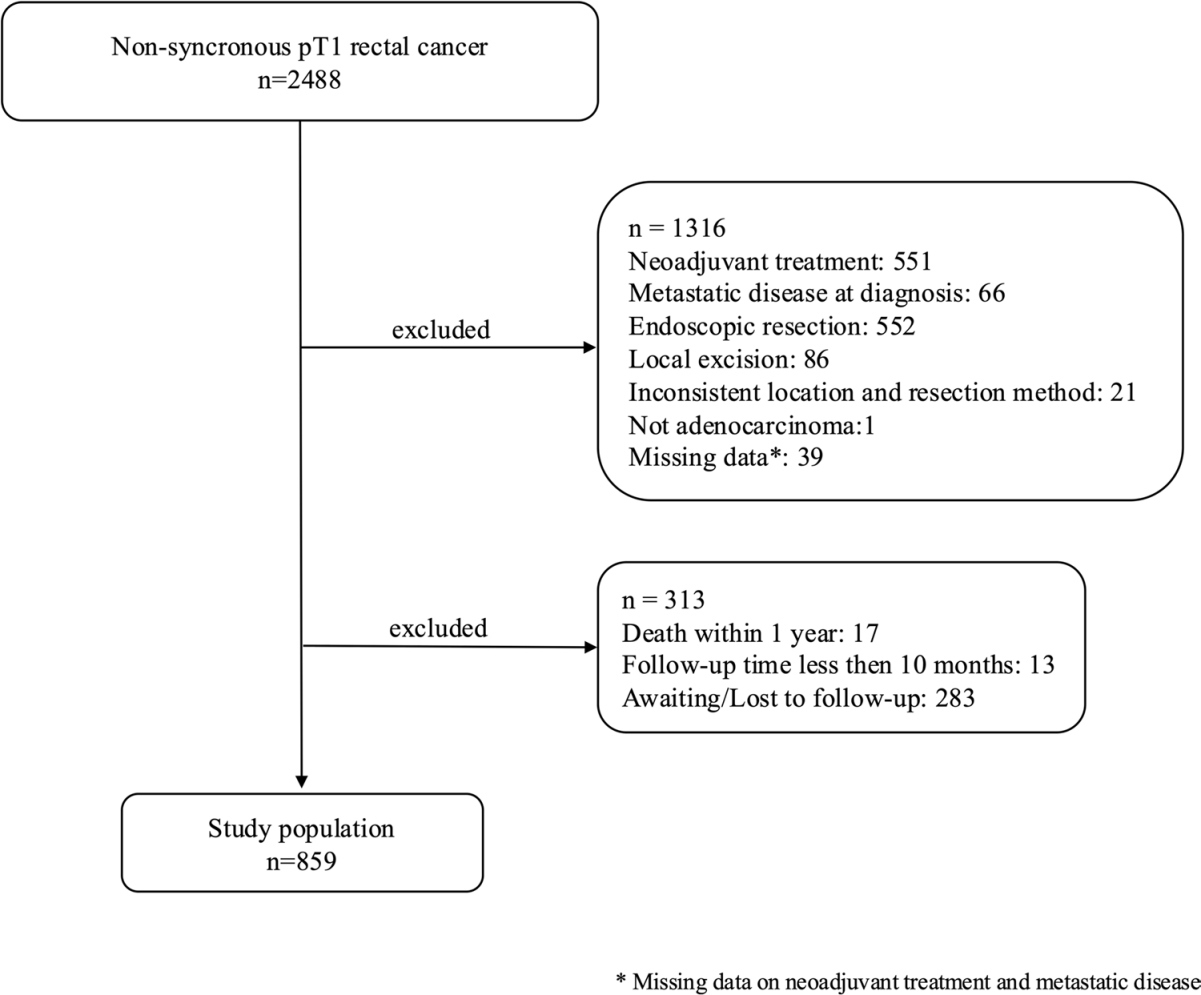
Flowchart illustrating the inclusion and exclusion process

Patients with surgically treated, non-synchronous pT1 RC diagnosed between January 2009 and December 2022 were identified in the SCRCR. Patients were excluded if they received neoadjuvant treatment, had distant metastases at index operation, non-adenocarcinoma histology, underwent undefined local excision or endoscopic resection, or if tumor location was inconsistent with surgical approach. Additionally, patients were excluded if they died within one year, had a follow-up time of less than 10 months (defined as incomplete 1-year follow-up), or were awaiting or lost to follow-up (Fig. 1).

### Exposures and potential confounding factors

Exposures in this study consist of TEM [19] versus conventional rectal resection procedures (high or low anterior resection, Hartmann’s procedure, or abdominoperineal resection). Potential confounding factors for recurrence included in this study were sex, age at diagnosis, American society of anesthesiologists (ASA) classification, histologic grade, LVI, perineural invasion (PNI), mucinous subtype, submucosal invasion depth, and R1/Rx resection. Age at diagnosis was kept as a continuous variable in the multivariable analyses. ASA classification was dichotomized into 1–2 and 3–4 and used in both imputational and multivariable analyses. Histologic grade was classified according to the WHO 2019 classification into low-grade or high-grade RC [20, 21]. Submucosal invasion was categorized according to Kikuchi/Kudo classification (Sm1–Sm3) [22, 23]. For imputational analyses and adjusted Cox proportional hazard regression, the variable was dichotomized into shallow (Sm1) and deep submucosal invasion (Sm2–Sm3). Resection margin assessment included both lateral and vertical dimensions with R0 defined as absence of tumor cells at the resection borders and Rx as non-assessable resection margins. Tumor size and tumor budding were not recorded in SCRCR during the study period and were therefore not included in analyses.

### Low- and high-risk cancers

ESGE classifies endoscopically resected tumors into high- and low-risk groups based on risk of LNM. High-risk tumors in this study were defined as exhibiting at least one of the following features: LVI, high histological grade, Sm2–3, or R1/Rx in accordance with current guidelines [9]. As tumor budding was not recorded in the SCRCR during the study period, it could not be included in the risk categorization. Patients with tumors lacking all high-risk features were categorized as low risk. Tumors for which low-risk categorization was precluded due to missing data were defined as indeterminate. For logistic regression analyses, missing values were imputed, enabling all patients to be categorized as either high or low risk.

### Outcomes

The primary outcome measure was overall recurrence, subdivided into local and distant recurrence. Local recurrence was defined as tumor in the anastomosis, in the adjacent rectal segment, or mesorectal lymph nodes, or, in cases of resection surgery, tumor in the tissue bordering the original operation site. Overall recurrence between TEM and surgical resection was compared using overall recurrence rate (%), 3- and 5-year disease-free interval (DFI), and estimated hazard ratios (HRs), both unadjusted and adjusted. Time at risk was calculated from the date of surgical intervention to the date of recurrence. Patients were censored at last follow-up, death, or migration within a 6-year follow-up period. The 6-year time cut-off was applied to account for potential delays in 5-year follow-up appointments. In risk group analyses, five patients experienced both local and distant recurrences and were therefore represented in both the local and distant recurrence groups.

### Missing data

Multiple imputation was carried out in R using the Multiple Imputation by Chained Equations (MICE) package [24]. The plausibility of a missing-not-at-random (MNAR) mechanism was visually assessed using missingness pattern matrices. The plausibility of the MAR assumption was evaluated through clinical reasoning and by examining associations between missingness and relevant observed variables. Associations for categorical variables and continuous variables were assessed using chi-square test and correlation analyses, respectively. Variables included in the MI model are shown in Supplementary Table 2, and in addition the Nelson–Aalen estimator was included. MI was performed at the variable level and risk groups were constructed after individual variables had been imputed. The maximum number of iterations was set to 100, and 50 imputations were performed. Convergence of the fully conditional specification algorithm was evaluated using trace plots of the mean and standard deviation of imputed variables across iterations, which showed stability of the imputation chain. Density plots were used to compare imputed and observed values, supporting the plausibility of the imputations. For hypothesis testing, the imputed results are presented as primary. For comparison, sensitivity analyses using complete-case data were performed and are presented separately both in result section and Supplementary Tables.

### Statistical analyses

A statistical analysis plan was established during study design, although not formally documented. The only deviation from the plan is reported below. Analyses were conducted in IBM SPSS version 29 and R (version 4.4.2; R Core Team, 2024) [25, 26]. A 95% confidence interval (CI) was used and *p* values of < 0.05 were considered statistically significant. Quantitative variables were described with median and IQR. Differences in follow-up time were tested using the Mann–Whitney *U* test and difference in proportions using Chi-square test and Fisher’s exact test when appropriate. Three- and five-year DFI was calculated using Kaplan–Meier analyses and compared with log-rank tests. To estimate the risk of overall recurrence, adjusted Cox proportional hazards regressions were performed. For assessment of the proportional hazard assumption, Kaplan–Meier and log (− log) plots were generated in addition to Schoenfeld residual tests. The proportionality was considered met for all potential confounders except histologic grade. Therefore, the Cox proportional hazards regression comparing overall recurrence between surgical resection and TEM were adjusted for sex, age at diagnosis, ASA class, LVI, PNI, mucinous subtype, submucosal invasion, and resection margin. Sensitivity analysis was performed including histologic grade. As the stratification by risk group is based on the pathological risk factors for LNM in guidelines, logistic regression models were further adjusted for the remaining clinical potential confounders: sex, age at diagnosis, and ASA classification. For risk-group analyses, logistic regression was used rather than an initially planned Cox regression model due to violation of the proportional hazards assumption, likely related to low EPV ratios in these subgroups.

### Ethical considerations

The study was prior to start approved by the Swedish ethical review authority board (2023-01159-01) and was carried out in accordance with the Helsinki declaration. All data were coded to ensure patient anonymity.

## Results

### Patient and tumor characteristics

A total of 2488 patients with pT1 RC were identified in the registry. Of these, 1316 patients were excluded, leaving 859 patients in the final study population (Fig. 1). TEM was performed in 228 patients, and 631 patients underwent surgical resection. The median follow-up time was 59.7 months (38.5–62.3), with no statistical difference between the groups.

An overview of baseline clinicopathological factors is shown in Table 1. There was no substantial difference in distribution of sex, LVI, PNI, or mucinous subtype, between the groups. Patients in the TEM group were older and had more comorbidities, and a higher proportion of Sm1 tumors, low histologic grade, and R1/Rx resections compared with the surgical group. In the surgical group, 12% of the patients had concomitant LNM.

**Table 1:** Distribution of clinicopathological factors in patients who underwent surgical resection or TEM

		Surgical resection	TEM
		*n* (%)	*n* (%)
	Total *n*	631	228
Sex			
Male	486	354 (56.1)	132 (57.9)
Female	373	277 (43.9)	96 (42.1)
Age at diagnosis			
Median [IQR]		69 [61–75]	72 [66–79]
ASA			
1	162	119 (19.0)	43 (19.5)
2	463	366 (58.6)	97 (44.1)
3	214	137 (21.9)	77 (35.0)
4	6	3 (0.5)	3 (1.4)
Missing	14	6	8
Histologic grade			
Low grade	726	546 (93.7)	180 (97.3)
High grade	42	37 (6.3)	5 (2.7)
Missing	91	48	43
Lymphovascular invasion			
No	640	526 (88.9)	114 (89.1)
Yes	80	66 (11.1)	14 (10.9)
Missing	139	39	100
Perineural invasion			
No	680	568 (97.6)	112 (100)
Yes	14	14 (2.4)	0
Missing	165	49	116
Mucinous subtype			
No	736	573 (95.8)	163 (95.3)
Yes	33	25 (4.2)	8 (4.7)
Missing	90	33	57
Submucosal invasion			
Sm1	222	133 (26.9)	89 (46.1)
Sm2	165	119 (24.1)	46 (23.8)
Sm3	300	242 (49.0)	58 (30.1)
Missing	172	137	35
N stage			
N0		546 (87.9)	–
N +		75 (12.1)	–
Missing		10	
Resection margin			
R0	784	603 (97.1)	181 (81.9)
R1	19	10 (1.6)	9 (4.1)
Rx	39	8 (1.3)	31 (14.0)
Missing	17	10	7

### Recurrence

**Fig. 2:**
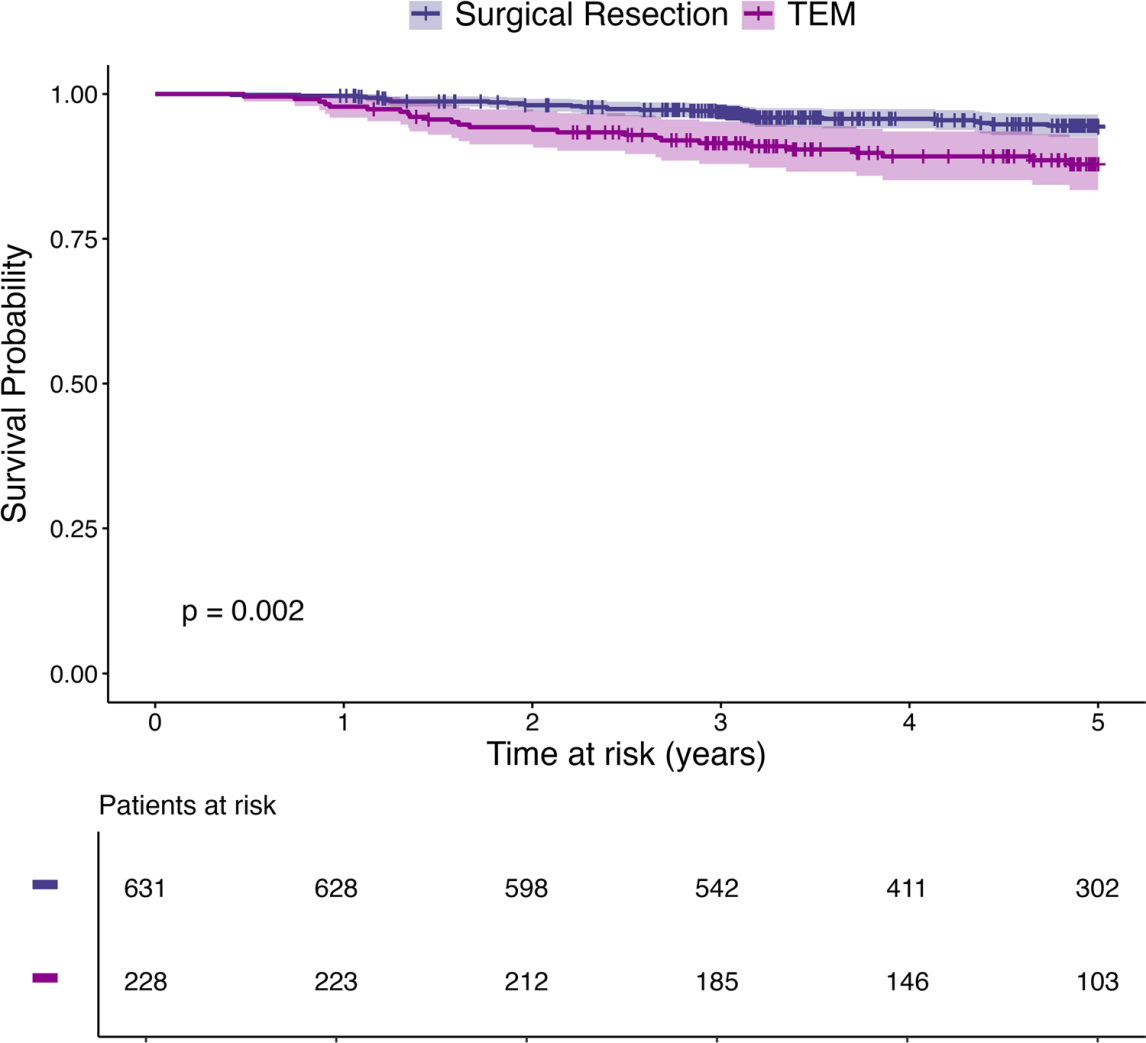
Kaplan–Meier curve illustrating recurrences over time according to treatment group

The proportion of overall recurrence was 11.0% (25/228) in patients treated with TEM, which was higher than the 4.9% (31/631) observed after surgical resection (*p* = 0.002). Patients undergoing surgical resection primarily experienced distant recurrences, in contrast to the TEM group, in which local recurrences predominated (Table 2). There were significantly more local recurrences in the TEM group compared to surgical resection (*p* < 0.001), while no difference was found in distant recurrences. Out of 18 patients who experienced isolated local recurrence, 6 (33%) died during follow-up. Median time to overall recurrence was 20 months (16–35) after TEM and 29 months (18–38) after surgical resection (*p* = 0.232). Three- and five-year DFIs were 92% (95% CI: 88–95) and 88% (95% CI: 83–93) after TEM, respectively, which was significantly lower than 97% (95% CI: 96–98) and 95% (95% CI: 93–97), after surgical resection (log-rank test,* p* = 0.002) (Fig. 2). The unadjusted HR of 2.28 (95% CI: 1.34–3.85) and the adjusted HR (imputed data) of 2.83 (95% CI: 1.56–5.13; *p* = 0.001) both show significantly higher overall recurrence following TEM.

**Table 2:** Proportion of local and distant recurrences according to resection method

		Surgical resection	TEM	
		*n* (%)	*n* (%)	
Total *n* recurrences	56	31/631 (4.9)	25/228 (11.0)	*p* value
Local	18	4 (0.6)	14 (6.2)	< 0.001
Distant	32	25 (4.0)	7 (3.1)	0.542
Local + distant	5	2 (0.3)	3 (1.3)	0.089
Missing location	1	0	1	–

### Risk groups

As presented in Table 3, the overall recurrence in the low-risk group was higher after TEM (11.1%) compared to surgical resection (1.9%) (*p* = 0.039), with logistic regression showing a fivefold higher OR for the TEM group (OR 5.31, 95% CI: 1.10–25.56; *p* = 0.039) (Table 4). In the high-risk group, the overall recurrence rate after TEM was 11.8% compared to 6.5% following surgical resection (*p* = 0.052) with an OR of 2.39 (95% CI: 1.22–4.66; *p* = 0.011). There were significant differences in local recurrence rates, in both the low- and high-risk groups between TEM and surgical resection (Table 5). In the low-risk group, 8.3% of patients in the TEM group experienced a local recurrence, whereas no surgically treated patient did (*p* = 0.016). Due to the absence of local recurrences in the surgical low-risk group, a stable logistic model could not be fitted to compare TEM and surgical resection in this specific group. Among high-risk patients, local recurrence occurred in 7.9% of those undergoing TEM versus 1.3% in the surgical resection group (*p* < 0.001) with an OR of 6.96 (95% CI: 2.40–20.17; *p* < 0.001).

**Table 3:** Overall recurrence rates by risk groups

		Recurrence	
	Total *n*:	% (*n*)	*p* value
**Low risk**			
Surgical resection	103	1.9% (2)	0.039
TEM	36	11.1% (4)	
**High risk**			
Surgical resection	400	6.5% (26)	0.052
TEM	127	11.8% (15)	
**Indeterminate risk**			
Surgical resection	128	2.3% (3)	0.063
TEM	65	9.2% (6)	

Low risk: low histologic grade, absence of LVI, Sm1, and R0-resected tumors

High risk: at least one of high grade, LVI, Sm > 1, or R1/Rx

Indeterminate risk: low-risk classification precluded due to missing data

**Table 4:** Logistic regression on imputed data, evaluating recurrence according to high- and low-risk groups

	Unadjusted			Adjusted		
	OR	95% CI	*p* value	OR	95% CI	*p* value
**Low risk**						
Overall recurrence						
Surgical resection	1.00	Ref		1.00	Ref	
TEM	5.97	1.29–27.69	0.024	5.31	1.10–25.56	0.039
Local recurrence						
Surgical Resection	–	–	–	–	–	–
TEM						
Distant recurrence						
Surgical resection	1.00	Ref		1.00	Ref	
TEM	2.91	0.50–16.73	0.234	2.82	0.44–18.01	0.275
**High risk**						
Overall recurrence						
Surgical resection	1.00	Ref		1.00	Ref	
TEM	2.11	1.11–4.03	0.024	2.39	1.22–4.66	0.011
Local recurrence						
Surgical resection	1.00	Ref		1.00	Ref	
TEM	7.15	2.54–20.15	< 0.001	6.96	2.40–20.17	< 0.001
Distant recurrence						
Surgical resection	1.00	Ref		1.00	Ref	
TEM	0.82	0.32–2.10	0.682	0.95	0.37–2.47	0.917

Adjusted analyses include the following potential confounding clinical factors: age at diagnosis, sex, and ASA classification

**Table 5:** Local recurrence rates by risk groups

		Recurrence	
	Total *n*:	% (*n*)	*p* value
**Low risk**			
Surgical resection	103	0	0.016
TEM	36	8.3% (3)	
**High risk**			
Surgical resection	400	1.3% (5)	< 0.001
TEM	127	7.9% (10)	
**Indeterminate risk**			
Surgical resection	128	0.8% (1)	0.045
TEM	65	6.2% (4)	

Low risk: low histologic grade, absence of LVI, Sm1, and R0-resected tumors

High risk: at least one of high grade, LVI, Sm > 1, or R1/Rx

Indeterminate risk: low-risk classification precluded due to missing data

Five patients who experienced both local and distant recurrences are represented in both Tables 5 and 6

No significant differences in distant recurrences were observed between TEM and surgical resection across the different risk groups (Table 6).

**Table 6:** Distant recurrence rates by risk groups

		Recurrence	
	Total *n*:	% (*n*)	*p* value
**Low risk**			
Surgical resection	103	1.9% (2)	0.572
TEM	36	5.6% (2)	
**High risk**			
Surgical resection	400	5.8% (23)	0.427
TEM	127	3.9% (5)	
**Indeterminate risk**			
Surgical resection	128	1.6% (2)	0.337
TEM	65	4.6% (3)	

Low risk: low histologic grade, absence of LVI, Sm1, and R0-resected tumors

High risk: at least one of high grade, LVI, Sm > 1, or R1/Rx

Indeterminate risk: low-risk classification precluded due to missing data

Five patients who experienced both local and distant recurrences are represented in both Tables 5 and 6

To account for time, Kaplan–Meier curves, corresponding life tables, and log-rank test were performed, showing results consistent with aforementioned analyses (Supplementary Fig. 1a–f).

When analyzing TEM separately, no significant difference in recurrence was observed between low- and high-risk tumors in either overall (11.1 vs. 11.8%; *p* = 0.999) or local recurrence (8.3 vs. 7.9%; *p* = 0.999). Of the five patients who experienced both local and distant recurrence, three had tumors with high-risk features, one had low-risk, and one was in the indetermined-risk group.

### Missing data and sensitivity analyses

Most missing data were present in Sm classification (20.0%), PNI (19.2%), LVI (16.2%), histologic grade (10.6%), and mucinous subtype (10.2%). The missingness pattern matrices showed a non-monotone, irregular, and scattered pattern. No large clusters or horizontal or vertical bands suggestive of MNAR were observed. Three auxiliary variables that predicted missingness were added to the multiple imputation model (Supplementary Table 2). In addition, several variables already included for the planned hypothesis-testing analysis also predicted missingness. As a sensitivity analysis, comparisons of overall recurrence between TEM and surgical resection were repeated using complete-case data. This resulted in the exclusion of 37% of patients and 24 events in the adjusted model. In the complete-case adjusted analysis, the HR was 2.23 (95% CI: 0.90–5.50). The estimated HRs were somewhat smaller and their 95% CIs wider compared with the imputed data analyses, indicating correction of bias and increased power from multiple imputation. Sensitivity logistic regression risk-group analyses based on complete-case data are presented in Supplementary Table 3.

## Discussion

This nationwide registry-based study demonstrates that local recurrence occurs more frequently following TEM compared to surgical resection, not only in high- but also in low-risk T1 RC patients . Notably, current guidelines are exclusively based on risk of concomitant LNM. Thus, our novel findings highlight that considering risk of local recurrence is likely critical when deciding treatment for T1 RC patients.

In this study, local recurrence rate was significantly higher after TEM compared to surgical resection, which is in line with earlier studies [11–14]. When stratified on risk groups defined according to ESGE guidelines, local recurrence rate was significantly higher after TEM compared with surgical resection in the high-risk group (8 vs. 1%), and notably also in the low-risk group (8 vs. 0%). In fact, this is the first study comparing local recurrence rates between TEM and surgical resection, stratified by high- and low-risk groups according to current risk criteria. These findings are particularly valuable, as no additional treatment is typically considered necessary for patients with low-risk tumors.

In addition, when comparing local recurrence rates between low- and high-risk groups following TEM alone (8.3 vs. 7.9%), no statistically significant difference was observed, raising doubts about the utility of the current risk categorization after TEM. In line with our results, Doornebosch et al. demonstrated no difference in local recurrence rates between low- and high-risk groups following TEM. Of note, the local recurrence rates in their study were higher than observed in the present study, 30 and 33% in the low- and high-risk groups, respectively [27]. In contrast, some studies have demonstrated a difference in local recurrence across risk groups following TEM [16, 28, 29]. What is evident in these studies is that patients with high-risk tumors experienced higher rates (> 30%) of local recurrence compared to the present study (8%), and the range of local recurrence rates among patients with low-risk tumors was wide (4.3–30%) [16, 27–29]. This variability may be attributed to the studies being underpowered and to differences in how risk groups were defined [27–30].

Higher local recurrence rates after TEM may be explained by incomplete resections, a well-known disadvantage of using TEM [11]. Consistent with earlier studies, R1/Rx resections were more frequently observed in patients undergoing TEM (18%), than after surgical resection (3%) [16, 31–34]. Notably, the increase in local recurrence rate was also observed in the low-risk group, which, by definition, had tumors removed with clear margins (R0). The reason why low-risk patients nonetheless experience high rates of local recurrences after TEM remains unclear. One explanation relates to the properties of the TEM technique, whereby manipulation of the tumor may cause the release of free cancer cells, which can subsequently reattach to the resection site and later develop into a local recurrence. A key technical distinction between TEM and other local resection techniques, such as endoscopic submucosal dissection (ESD), is that TEM extends the resection entirely through the rectal wall and into the perirectal fat [19]. Consequently, important protective oncological barriers, such as muscularis propria, that otherwise would help prevent spread of loose tumor cells into adjacent pelvic tissue are breached. These factors may explain the tendency toward higher recurrence rates following TEM compared to other endoscopic resection techniques [30, 35].

Undiagnosed concomitant LNM may be another contributor to the increased local recurrence rates observed after TEM, stemming from inaccuracies in preoperative MRI evaluation [36]. However, the low-risk ESGE criteria should predict low risk for concomitant LNM. Therefore, residual LNM is unlikely to explain the similar local recurrence rates observed in the low- and high-risk groups following TEM (8.3 vs. 7.9%), nor the difference in local recurrence between TEM and surgically resected patients classified as low risk (8 vs. 0%). In this context, it is interesting to note that Wetterholm et al. observed a local recurrence rate of 32% after TEM treatment versus 1% following surgical resection in T2 RC [37], supporting the notion that TEM is indeed associated with high risk of local recurrence in early RC. Finally, although salvage surgery is often feasible after local recurrence, and R0 resection [38] most often achieved, patients with local recurrences nevertheless experience substantially decreased survival and reduced quality of life [39–41]. In light of these findings, the ESGE treatment recommendations based on risk group appear less applicable following local resection with TEM. One might even question whether TEM should be used with curative intent in T1 RC, given the high risk of local recurrences, even among low-risk patients.

This study demonstrates a 2.8-fold higher risk of overall recurrence after TEM compared with surgical resection (11 vs. 5%). This finding aligns with two previous meta-analyses of early RC [11, 12], where the difference in overall recurrence appears to be generated by a higher local recurrence rate after TEM. The differences in overall recurrence between TEM and surgical resection remains after adjusting for R1/Rx, suggesting the difference is independent of R1/Rx resection. Across risk groups, overall recurrence in the low-risk group was five times more frequent after TEM compared to surgical resection (11 vs. 2%) and nearly doubled (12 vs. 7%) in the high-risk group. Only one earlier study was identified in the literature that explicitly examined overall recurrences after TEM and surgical resection, stratified on high- and low-risk groups [16]. However, that study defined low risk as the absence of high histologic grade and lymphatic invasion but did not consider R1/Rx resection. In fact, all patients with recurrences in the TEM ‘low-risk’ group in that study had histologically non-radical index resections, thereby classifying them as high-risk according to current guidelines, which precludes any meaningful comparison.

Moreover, this study found no difference in distant metastases between TEM and surgical resection, consistent with previously published meta-analyses [11, 12, 14], and this finding remained unchanged after stratification into risk groups. Studies exclusively investigating distant recurrence across risk groups are not available in the literature.

A common limitation of registry-based studies is selection bias. In this study, patients in the TEM group were older, had more comorbidities, and had tumors that were more often Sm1, of low histologic grade, or resected as R1/Rx to a greater extent. To reduce the impact of this imbalance, adjusted analyses were performed for all potential confounders except histologic grade, which was excluded due to non-proportional hazards. However, a sensitivity analysis including histologic grade as a potential confounder was conducted and demonstrated minimal changes in HR and corresponding 95% CI. In addition, patients were stratified into high- and low-risk groups based on variables including histologic grade, thereby accounting for this factor in the analyses.

Missing data constitutes another limitation. To reduce bias due to missingness and to enhance the power of the study, multiple imputation using MICE was performed. Missingness patterns showed a non-monotone, scattered distribution without systematic clustering, which would be atypical for MNAR. In addition, several observed variables predicted missingness, consistent with a plausible MAR assumption. The inclusion of the Nelson–Aalen estimator is known to support robust multiple imputation and was therefore incorporated into the imputation model [24, 42]. Diagnostics of the imputation procedure, including assessment of the convergence, were performed and indicated that the imputation process was valid. Hazard ratios were consistent across analyses; however, statistical significance was observed more frequently in the imputed dataset, likely reflecting greater statistical power compared with the complete-case analyses.

During the study period, LNM risk factors have gained increasing recognition, potentially influencing reporting. However, histologic grade, resection margin, LVI, and submucosal invasion depth were consistently registered in the SCRCR, thereby the risk of bias is likely modest.

Finally, tumor budding, size, and pedunculated morphology were not included in the SCRCR registry during the period of inclusion. The international tumor budding consensus conference concluded in 2016 that tumor budding (Bd2–3) is an independent predictor for LNM in pT1 CRC [43]. Lack of information about tumor budding is a limitation and may potentially lead to the misclassification of a true high-risk tumor into the low-risk group. Nonetheless, tumor budding (Bd2–3) is often associated with other unfavorable histopathological features, such as high histologic grade and LVI [44]. Moreover, recent studies have validated that poorly differentiated clusters (PDS) add prognostic value in CRC. However, as current guidelines were evaluated, and the study period spans since 2009, it was not achievable to consider PDS in the present study. Furthermore, a tumor size greater than 3 cm has been associated with higher local recurrence rates after TEM [27]. The lack of tumor size data may result in residual confounding, potentially reflecting confounding by indication. Patients with large tumors are more likely to be recommended surgical resection. Consequently, the observed difference in recurrence rates between TEM and surgical resection may be underestimated, and the true difference could be even greater if tumor sizes were accounted for. Moreover, pedunculated morphology, which has been associated with decreased recurrence [45], was not included in the SCRCR. As such lesions are extremely uncommon in the rectum, their influence on the overall findings of the present study is likely minor.

This registry-based study on prospectively collected data is strengthened by its nationwide coverage and an average completeness of 99% compared to the compulsory cancer registry in Sweden [18]. The SCRCR includes patients from all regions of Sweden, treated in both private and public hospitals, thereby mitigating potential selection bias associated with socioeconomic status. The follow-up time was considered sufficient to detect potential recurrences (median 59 months), as over 88% of the patients without recurrence were followed for three years or more, which is when most recurrences are typically detected.

Taken together, given that surgical resection is associated with a high complication rate and substantial morbidity, these risks must be weighed against the high local recurrence risk following TEM, which in this study was observed even in low-risk patients. This novel finding calls into question the use of TEM as a local resection technique for curative intent in pT1 RC. However, local resection offers several advantages over surgical resection, and other endoscopic resection techniques appear to be associated with more favorable local recurrence rates in low-risk groups. Therefore, these techniques may represent more suitable primary treatment options than TEM in cases where local resection is indicated.

In conclusion, this study on T1 RC demonstrates significantly higher local recurrence rates in patients undergoing TEM compared to surgical resection, even among those with low-risk tumors, where TEM may be considered definitive treatment. These findings question the use of TEM in the management of T1 RC with curative intent.

## Supplementary Information

Below is the link to the electronic supplementary material.

Supplementary file1 (TIFF 2529 KB)

Supplementary file2 (DOC 98 KB)

Supplementary file3 (DOCX 32 KB)

Supplementary file4 (DOCX 28 KB)
