# Supplementary material for: Transanal endoscopic microsurgery was associated with higher recurrence rate in both low- and high-risk T1 rectal cancer compared to surgical resection
Source: Surg Endosc. 2026 Jun 5;40(8):6585–95. doi: 10.1007/s00464-026-12926-y (PMC13457268; doi:10.1007/s00464-026-12926-y)
Supplement: Supplementary file 1 [file 464_2026_12926_MOESM3_ESM.docx]

Supplementary Table 2. Pre-imputational analyses to assess the data in preparation for constructing the imputation model. The columns show associations with variable missingness, associations with the variable itself, and indicate which variables are included in the analyses and which imputation model was used.

| **Variables** | **MAR plausibility** | | | | | | | | **Predicitve of variable** | | | | | | | **Analysis** | **Model** |
| --- | --- | --- | --- | --- | --- | --- | --- | --- | --- | --- | --- | --- | --- | --- | --- | --- | --- |
|  | LVI | PNI | HG | SM | MU | RM | ASA | Overall | LVI | PNI | HG | SM | MU | RM | ASA |  |  |
| Sex |  |  |  |  |  |  |  |  |  |  |  |  |  |  |  |  | - |
| Age at diagnosis |  |  |  |  |  |  |  |  |  |  |  |  |  |  |  |  | - |
| ASA classification |  |  |  |  |  |  |  |  |  |  |  |  |  |  |  |  | Log reg |
| Histologic grade |  |  |  |  |  |  |  |  |  |  |  |  |  |  |  |  | Log reg |
| LVI |  |  |  |  |  |  |  |  |  |  |  |  |  |  |  |  | Log reg |
| PNI |  |  |  |  |  |  |  |  |  |  |  |  |  |  |  |  | Log reg |
| Mucinous subtype |  |  |  |  |  |  |  |  |  |  |  |  |  |  |  |  | Log reg |
| Submucosal invasion |  |  |  |  |  |  |  |  |  |  |  |  |  |  |  |  | Log reg |
| Resection margin |  |  |  |  |  |  |  |  |  |  |  |  |  |  |  |  | Log reg |
| Time variabel |  |  |  |  |  |  |  |  |  |  |  |  |  |  |  |  | - |
| Recurrence (overall) |  |  |  |  |  |  |  |  |  |  |  |  |  |  |  |  | - |
| Recurrence (distant) |  |  |  |  |  |  |  |  |  |  |  |  |  |  |  |  | - |
| Recurrence (local) |  |  |  |  |  |  |  |  |  |  |  |  |  |  |  |  | - |
| Resection type |  |  |  |  |  |  |  |  |  |  |  |  |  |  |  |  | - |
| Vital status |  |  |  |  |  |  |  |  |  |  |  |  |  |  |  |  | - |
| Location of tumour |  |  |  |  |  |  |  |  |  |  |  |  |  |  |  |  | PMM |
| Adjuvant treatment |  |  |  |  |  |  |  |  |  |  |  |  |  |  |  |  | Log reg |

Dark green indicates a stronger association than light green.

Abbreviations: LVI, lymphovascular invasion; PNI, perineural invasion; HG, histologic grade; SM, submucosal invasion; MU, mucinous subtype: RM, resection margin; Log reg, logistic regression; PMM, predictive mean matching.

Adjuvant treatment refers to intended adjuvant oncological treatment.
