# Supplementary material for: Transanal endoscopic microsurgery was associated with higher recurrence rate in both low- and high-risk T1 rectal cancer compared to surgical resection
Source: Surg Endosc. 2026 Jun 5;40(8):6585–95. doi: 10.1007/s00464-026-12926-y (PMC13457268; doi:10.1007/s00464-026-12926-y)
Supplement: Supplementary file 2 [file 464_2026_12926_MOESM4_ESM.docx]

|  |  |  | Unadjusted | | |  | Adjusted | | |
| --- | --- | --- | --- | --- | --- | --- | --- | --- | --- |
|  |  |  | OR | 95% CI | *p*-value |  | OR | 95% CI | *p*-value |
|  |  |  |  |  |  |  |  |  |  |
| Low-risk |  |  |  |  |  |  |  |  |  |
|  |  |  |  |  |  |  |  |  |  |
| Overall recurrence |  | Surgical Resection | 1.00 | Ref. |  |  | 1.00 | Ref. |  |
|  |  | TEM | 6.31 | 1.10–36.08 | 0.038 |  | 4.99 | 0.82–30.48 | 0.082 |
|  |  |  |  |  |  |  |  |  |  |
| Local Recurrence |  | Surgical Resection |  |  |  |  |  |  |  |
|  |  | TEM | - | - | - |  | - | - | - |
|  |  |  |  |  |  |  |  |  |  |
| Distant recurrence |  | Surgical Resection | 1.00 | Ref. |  |  | 1.00 | Ref. |  |
|  |  | TEM | 2.91 | 0.50–16.73 | 0.234 |  | 2.82 | 0.44–18.01 | 0.275 |
|  |  |  |  |  |  |  |  |  |  |
| High-risk |  |  |  |  |  |  |  |  |  |
|  |  |  |  |  |  |  |  |  |  |
| Overall recurrence |  | Surgical Resection | 1.00 | Ref. |  |  | 1.00 | Ref. |  |
|  |  | TEM | 1.93 | 0.99–4.45 | 0.055 |  | 2.18 | 1.07–4.45 | 0.032 |
|  |  |  |  |  |  |  |  |  |  |
| Local Recurrence |  | Surgical Resection | 1.00 | Ref. |  |  | 1.00 | Ref. |  |
|  |  | TEM | 6.75 | 2.26–20.15 | <0.001 |  | 6.35 | 2.01–20.06 | 0.002 |
|  |  |  |  |  |  |  |  |  |  |
| Distant recurrence |  | Surgical Resection | 1.00 | Ref. |  |  | 1.00 | Ref. |  |
|  |  | TEM | 0.67 | 0.25–1.80 | 0.430 |  | 0.84 | 0.31–2.31 | 0.735 |
|  |  |  |  |  |  |  |  |  |  |

Supplementary Table 3. Sensitivity analyses using complete-case data corresponding to Table 4.
