# Supplementary material for: Transanal endoscopic microsurgery was associated with higher recurrence rate in both low- and high-risk T1 rectal cancer compared to surgical resection
Source: Surg Endosc. 2026 Jun 5;40(8):6585–95. doi: 10.1007/s00464-026-12926-y (PMC13457268; doi:10.1007/s00464-026-12926-y)
Supplement: Supplementary file 3 [file 464_2026_12926_MOESM2_ESM.doc]

STROBE Statement—Checklist of items that should be included in reports of ***cohort studies***

|  | Item No | Recommendation |
| --- | --- | --- |
| **Title and abstract** | 1 | (*a*) Indicate the study’s design with a commonly used term in the title or the abstract.  ***Abstract – Methods – first sentence.*** |
|  |  | (*b*) Provide in the abstract an informative and balanced summary of what was done and what was found  ***Abstract – 242 words including headings. Background, main methods, main results and conclusion are accounted for.*** |
| Introduction | | |
| Background/rationale | 2 | Explain the scientific background and rationale for the investigation being reported  ***Introduction – The introduction is a summary of the present state of research in the field and explains the rationale behind the importance of conducting this study.*** |
| Objectives | 3 | State specific objectives, including any prespecified hypotheses  ***Introduction – the objectives is stated in the end of the introduction.*** |
| Methods | | |
| Study design | 4 | Present key elements of study design early in the paper  ***Material and Methods – Study design. The design is clearly stated.*** |
| Setting | 5 | Describe the setting, locations, and relevant dates, including periods of recruitment, exposure, follow-up, and data collection  ***Material and Methods – Swedish Colorectal Cancer Registry:***  ***– The settings: Nationwide registry study.***  ***–Follow-up: 1, 3 and 5 years postoperatively.***  ***Material and Methods – Study population***  ***–Recruitment period.***  ***–Data collection specifics.***  ***Material and Methods – Exposures and potential confounders***  ***–Exposure***  ***Material and Methods – Outcome***  ***–Follow-up period and cut off*** |
| Participants | 6 | (*a*) Give the eligibility criteria, and the sources and methods of selection of participants. Describe methods of follow-up  ***Material and Methods – Study population***  ***–Inclusion and exclusion criteria stated.***  ***–Figure 1. Flow-chart of the inclusion/exclusion process.***  ***Material and Methods – Swedish Colorectal Cancer Registry***  –***Methods of follow-up described for both surgical resection and TEM.*** |
|  |  | (*b*) ~~For matched studies, give matching criteria and number of exposed and unexposed~~  ***NA*** |
| Variables | 7 | Clearly define all outcomes, exposures, predictors, potential confounders, and effect modifiers. Give diagnostic criteria, if applicable  ***Material and Methods – Exposures and potential confounding factors –***  ***– Exposures and all potential confounders that are included in multivariable analyses are described in this section.***  ***Material and Methods***  ***–Outcome – The outcomes and diagnostic criteria (overall, local and distant recurrence) are explained in the first part of this section. Further the outcome measures are explained.*** |
| Data sources/ measurement | 8* | For each variable of interest, give sources of data and details of methods of assessment (measurement). Describe comparability of assessment methods if there is more than one group.  ***Material and Methods – Exposures and potential confounding factors***  ***All variables of interest that were further used as potential confounder or were a part of the risk stratification was described in this section.*** |
| Bias | 9 | Describe any efforts to address potential sources of bias  *Material and Methods ­– Missing values*  ***– Missing values are described and if left unhandled, could lead to selection bias.***  ***Material and Methods ­– Statistical analyses***  ***– Stratification into risk-groups is part of the primary aim, it reduces bias that is due to a skewed distribution of the potential histopathological confounders (LVI, histological grade, submucosal invasion and R1/Rx resection)*** |
| Study size | 10 | Explain how the study size was arrived at  ***Figure 1 – Flow chart*** |
| Quantitative variables | 11 | Explain how quantitative variables were handled in the analyses. If applicable, describe which groupings were chosen and why  ***Material and Methods ­– Exposure and potential confounding factors***  ***–The quantitative variable use is age at diagnosis, explanation in this section.*** |
| Statistical methods | 12 | (*a*) Describe all statistical methods, including those used to control for confounding  ***Material and Methods ­– Statistical analyses – including Cox regression*** |
|  |  | (*b*) Describe any methods used to examine subgroups and interactions  ***Material and Methods ­– Statistical analyses – Chi square test, Fishers exact test and logistic regression.*** |
|  |  | (*c*) Explain how missing data were addressed  ***Material and Methods ­***  ***– Missing values section.***  ***– Supplementary Table 2*** |
|  |  | (*d*) If applicable, explain how loss to follow-up was addressed  ***Material and Methods***  ***­– Study population section.***  ***–Figure 1.***  ***Material and Methods***  ***– Outcome – Patients were censored at last follow-up, death, or migration within a 6-year follow-up period*** |
|  |  | (*e*) Describe any sensitivity analyses  ***Material and Methods – Missing values – Last sentence.***  ***Supplementary table 3*** |
| Results | | |
| Participants | 13* | (a) Report numbers of individuals at each stage of study—eg numbers potentially eligible, examined for eligibility, confirmed eligible, included in the study, completing follow-up, and analysed  ***Results ­– Patients and tumour characteristics***  ***Figure 1 – Flow chart*** |
|  |  | (b) Give reasons for non-participation at each stage  ***Results ­– Patients and tumour characteristics and Figure 1.*** |
|  |  | (c) Consider use of a flow diagram  ***Figure 1 – Flow chart*** |
| Descriptive data | 14* | (a) Give characteristics of study participants (eg demographic, clinical, social) and information on exposures and potential confounders  ***Results ­– Patients and tumour characteristics***  ***Table 1*** |
|  |  | (b) Indicate number of participants with missing data for each variable of interest  ***Table 1***  ***Results ­– Missing data and complete case analyses***  ***Material and Methods ­– Missing values*** |
|  |  | (c) Summarise follow-up time (eg, average and total amount)  ***Results ­– Patients and tumour characteristics*** |
| Outcome data | 15* | Report numbers of outcome events or summary measures over time  ***Results ­– Recurrence – proportions and DFI are presented.***  ***Table 2*** |
| Main results | 16 | (*a*) Give unadjusted estimates and, if applicable, confounder-adjusted estimates and their precision (eg, 95% confidence interval). Make clear which confounders were adjusted for and why they were included  ***Results ­– Recurrence – last part of the section states the potential confounders.***  ***Results ­– Missing values and complete cases – unadjusted and adjusted HR complete case analyses.***  ***Table 3***  ***Table 4*** |
|  |  | (*b*) ~~Report category boundaries when continuous variables were categorized~~  *NA* |
|  |  | (*c*) ~~If relevant, consider translating estimates of relative risk into absolute risk for a meaningful time period~~  *NA* |
| Other analyses | 17 | Report other analyses done—eg analyses of subgroups and interactions, and sensitivity analyses  ***Supplementary table 3***  ***Results ­– Risk-groups – recurrence TEM vs TEM.*** |
| Discussion | | |
| Key results | 18 | Summarise key results with reference to study objectives  ***Discussion – first section*** |
| Limitations | 19 | Discuss limitations of the study, taking into account sources of potential bias or imprecision. Discuss both direction and magnitude of any potential bias  ***Discussion – page 16, line 22 – page 17, line 22*** |
| Interpretation | 20 | Give a cautious overall interpretation of results considering objectives, limitations, multiplicity of analyses, results from similar studies, and other relevant evidence  ***Discussion*** |
| Generalisability | 21 | Discuss the generalisability (external validity) of the study results  ***Discussion – page 17, line 22–24.*** |
| Other information | | |
| Funding | 22 | Give the source of funding and the role of the funders for the present study and, if applicable, for the original study on which the present article is based  ***Titel page – Grant support – line 16-17.*** |

*Give information separately for exposed and unexposed groups.

**Note:** An Explanation and Elaboration article discusses each checklist item and gives methodological background and published examples of transparent reporting. The STROBE checklist is best used in conjunction with this article (freely available on the Web sites of PLoS Medicine at http://www.plosmedicine.org/, Annals of Internal Medicine at http://www.annals.org/, and Epidemiology at http://www.epidem.com/). Information on the STROBE Initiative is available at http://www.strobe-statement.org.
